# Perceptions of Improved Biomass and Liquefied Petroleum Gas Stoves in Puno, Peru: Implications for Promoting Sustained and Exclusive Adoption of Clean Cooking Technologies

**DOI:** 10.3390/ijerph14020182

**Published:** 2017-02-13

**Authors:** Jacqueline Hollada, Kendra N. Williams, Catherine H. Miele, David Danz, Steven A. Harvey, William Checkley

**Affiliations:** 1 Division of Pulmonary and Critical Care, School of Medicine, Johns Hopkins University, Baltimore, MD 21287, USA; jhollada@jhu.edu (J.H.); chooper5@jhmi.edu (C.H.M.); 2 Social and Behavioral Interventions Program, Department of International Health, Bloomberg School of Public Health, Johns Hopkins University, Baltimore, MD 21205, USA; Kendra.Williams@jhu.edu (K.N.W.); Steven.Harvey@jhu.edu (S.A.H.); 3 Biomedical Research Unit, A.B. PRISMA, Lima 32, Peru; ddanzc@gmail.com

**Keywords:** clean fuel, liquefied petroleum gas (LPG), biomass, improved cookstoves, household air pollution, clean cooking adoption, exclusive use

## Abstract

Many households in low- and middle-income countries cook with inefficient biomass-burning stoves, which cause high levels of household air pollution and threaten long-term health. Although clean stoves and fuels are available, uptake and consistent use has been low. Using observations and in-depth interviews, we assessed the attitudes, preferences, and beliefs about traditional versus liquefied petroleum gas (LPG) stoves in rural Puno, Peru. A total of 31 in-depth interviews were conducted with primary cooks and their families, health workers, community leaders, and improved stove contractors. Six in-home observations of meal preparation were also conducted. Six major barriers to consistent use of clean stoves were identified: (1) perceived differences in food taste and nutrition by stove type; (2) cooking niches filled by different stoves; (3) social norms related to cooking practices; (4) safety concerns; (5) comparative costs of using different stoves; and (6) lack of awareness and concern about long-term health risks. These findings suggest that to successfully reduce household air pollution, clean cooking programs and policies must consider the many factors influencing adoption beyond health, such as cost, taste, fears, and cultural traditions. These factors could be incorporated into community-based and national efforts to scale-up sustained and exclusive adoption of clean cooking.

## 1. Introduction

Household air pollution (HAP), occurring when biomass fuels such as wood, dung, crop residues, and charcoal are burned indoors, is a crucial yet challenging public health issue. Globally, approximately 2.8 billion people use biomass fuel to cook and heat their homes, resulting in an estimated 11,000 deaths daily and nearly 4.3 million premature deaths per year [1,2].

Burning biomass fuels releases smoke and various toxins, including carbon monoxide and particulate matter, which have been linked to various acute and chronic negative health outcomes [3]. In many low-resource countries and rural populations where biomass fuels are burned indoors on a daily basis, kitchen level concentrations of harmful pollutants are far above that of acceptable international standards [4]. The World Health Organization (WHO) Indoor Air Quality (IAQ) Guidelines recommend that average annual levels of fine particulate matter of 2.5 μm in size (PM_2.5_) need to be at or below 10 μg/m^3^ to achieve health benefits, but even the interim target of 35 μg/m^3^ has not yet been attained in most low- and middle-income countries (LMICs) [2]. Women and children face the highest exposure, given that women are typically responsible for cooking family meals and children often stay near their mothers [1].

Two types of cleaner cooking solutions have been proposed to reduce household air pollution: improved biomass-burning stoves that burn more cleanly and efficiently than traditional stoves, and stoves that burn clean fuels including liquefied petroleum gas (LPG), electricity, ethanol, and biogas. Recently, efforts are focusing more on promoting clean fuels, given that to date no improved biomass-burning stove has been able to achieve the WHO IAQ guidelines [2]. However, especially in resource-poor settings, exclusive adoption of clean cooking technologies is rare [5].

Research has shown that to meet the WHO IAQ guidelines, near complete replacement of traditional stoves with low-emission stoves is necessary [6]. This requires eliminating the common practice of stove stacking, i.e., the concurrent use of both traditional and improved stoves, which is common in rural populations worldwide [7]. Thus, reducing exposure to household air pollution not only requires technological advancement, but also significant changes to cooking behavior, norms, and preferences, including stove use, fuel use, and food preparation [8].

Research has begun to focus on factors influencing adoption and consistent use of clean cooking technologies [9,10]. A recent systematic review highlighted seven key domains influencing improved stove adoption, including fuel and technology, household and setting, knowledge and perceptions, financing, markets, regulations, and policies or programs [10]. Although financial constraints to purchasing and maintaining improved stoves are frequently reported as a barrier to adoption, exclusive use of improved stoves remains uncommon even when financial barriers are removed [11,12]. Moreover, the promise of health benefits and smoke reduction may not be sufficient to encourage adoption of clean cooking [13].

### Household Air Pollution in Peru

Household air pollution is a problem globally, but the issue is most urgent in LMICs where the majority of households cook with biomass fuel [14]. In Peru, household air pollution is the fifth leading risk factor for disease, with a disproportionate impact in rural areas where 90% of households use biomass fuels for cooking and heating [15,16]. Recognizing this burden, the government of Peru and several local non-governmental organizations (NGOs) have initiated efforts to reduce household air pollution. The Fondo de Inclusión Social Energético (FISE) subsidizes LPG gas tanks for poor families, by providing vouchers that cover half the cost of one 20 kg gas tank per month (though people must purchase their own LPG stove). The regional government in Puno also supports several programs that provide improved biomass-burning stoves to qualifying poor households, and national campaigns encourage households to install chimneys. However, most chimneys are homemade and do not significantly improve air quality [17].

In Puno, reliance on biomass fuels for cooking is high in rural areas (97%), but only 5% in urban areas [18]. Pollard et al. [17] found that rural households using biomass stoves had PM_2.5_ levels up to four times higher than the WHO IAQ guidelines, and the presence of a chimney did not change these results. A recent qualitative study conducted in the high-altitude region of Ayacucho, Peru identified several barriers and enablers to adoption of improved biomass-burning stoves that align well with other findings [9]. In the Ayacucho region, the traditional stove was described as a fundamental part of the customs and ancestral heritage [5]. Women reported preferring the taste of food cooked on traditional stoves and using the ash produced as a type of fertilizer. Furthermore, women appreciated being able to use fuel that did not require purchase (e.g., animal dung) because of their limited financial situation. Women complained about the smoke produced by their traditional stoves, but they did not view the smoke as a threat to their long-term health. Though women reported some frustration about the amount of work required to maintain their traditional stove and the fact that the smoke blackened their walls, they were generally unaware of other available cooking options. The study by Rhodes et al. [5] focused on the traditional stove and did not explore perceptions of improved cooking technologies.

The purpose of the current study was to explore cooking norms and preferences influencing the adoption of LPG stoves and locally improved cookstoves in Puno, Peru, identify potential strategies to overcome clean cooking adoption barriers, and encourage more widespread exclusive use of clean cooking technologies. The results will also be used to inform strategies for promoting exclusive adoption of LPG within a multi-country randomized controlled intervention trial that aims to evaluate the impact of LPG use on household air pollution and the health of women and children in Peru, India, Guatemala, and Rwanda [19].

## 2. Materials and Methods

### 2.1. Study Setting

Puno is a region in southwestern Peru, located on the shore of Lake Titicaca at an altitude of 12,556 feet above sea level. A major hub for indigenous people of the Southwestern Andes, Puno is a rapidly growing low-resource setting with many of its Native-American Aymara and Quechua residents living in the plains surrounding the city. Rural dwellers in Puno live as subsistence farmers and cook almost exclusively indoors, due to continuous low temperatures throughout the year. Most cooking is done by women, once in the morning and once in the evening, using traditional stoves that burn combinations of wood, animal dung, and crop residue. Typically, the kitchen is built as a separate building next to the main living area [17].

**Figure 1:**
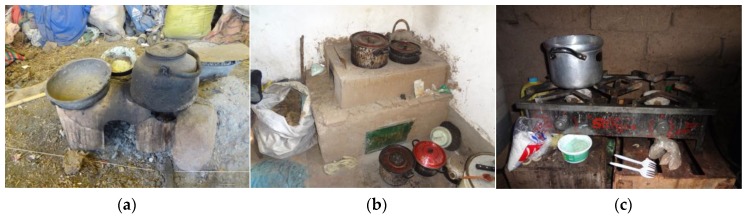
(**a**) Traditional, open-fire, biomass burning stove (*fogón*); (**b**) Locally improved, biomass burning stove (with a chimney on the building’s exterior); (**c**) Liquefied Petroleum Gas (LPG) stove.

Three types of stoves are used in the rural region: (1) traditional, open-fire, biomass-burning stoves (known as a *fogón* in Spanish or *q’uncha* in Aymara), (2) locally-made improved biomass-burning stoves, and (3) LPG stoves (Figure 1). Traditional stoves are typically constructed out of a mixture of mud, animal dung, and crop residues, have three burners, and burn any type of biomass fuel. Locally improved stoves seek to reduce stove emissions by better enclosing the fire and connecting a chimney to funnel the smoke outside. Locally improved stoves are typically built in the corner of the hut out of metal and the same mud mixture as traditional stoves, have three burners, and can burn all types of biomass fuels. Though these improved stoves reduce the visible amount of smoke in homes, they do not significantly reduce PM_2.5_ compared to the traditional open-fire stoves [20]. The LPG stoves commonly available and affordable in Puno are made from metal, have two burners, and attach to a refillable LPG tank; at the time of the study, four-burner models were also available but unaffordable for most rural residents, and three-burner models were not commercially available.

### 2.2. Data Collection

Data for this study were collected through qualitative in-depth interviews and participant observations from August to December 2015. In-depth interviews were conducted by three Spanish- and Aymara-speaking women from Puno, Peru who were trained in qualitative research methods. Observations were conducted by two qualitatively-trained graduate students. Participants were recruited using maximum variation sampling to ensure representation of various communities and perspectives, including people of different ages, income, and overall health. The sampling frame was designed to understand differences and similarities between household primary cooks and their family members, community leaders, improved stove contractors (individuals in the community who both sell and construct improved stoves), and health workers. We included primary cooks from households using different stoves and combinations of stoves (traditional, locally improved, and LPG). Improved stove contractors, community leaders, and health workers were identified and recruited through community contacts. Sample size was determined based on saturation; we ceased recruitment when the information observed and reported by participants became redundant and no new themes were emerging. Verbal informed consent was obtained from participants prior to conducting the interviews and observations.

Interviews were conducted in participants’ homes, in Aymara or Spanish depending on interviewee preference. The semi-structured interview guide explored participant perspectives on the three types of stoves, participant roles in the household and community, cooking practices, stove use patterns, fuel use, and perceptions of how cooking affects community, household, and individual health. All interviews were audio recorded, transcribed into Spanish, and translated into English. Translations of coded quotes were reviewed by a second translator to ensure accuracy.

Observations were conducted to contextualize the information reported during interviews. Field data collectors were trained to observe the participant’s cooking practices, including stove usage, utensils, food preparation, and interaction with other members of the household during meal preparation. Notes taken during field visits were written in English and expanded into observation transcripts within 24 h of each observation.

### 2.3. Data Analysis

Data analysis was structured into two rounds. First, researchers reviewed all transcripts to identify emerging themes, or issues that were mentioned by multiple people from different perspectives and referenced in both observations and interviews. A codebook was developed to summarize and describe each of the themes. In the second round of analysis, ATLAS.ti version 7 (Scientific Software Development GmbH, Berlin, Germany) [21] was used to code the transcripts based on the codebook. Researchers also wrote memos to summarize important information from the transcripts. The research team then reviewed the memos and the coded quotes, synthesized the information, compared within and across themes, and assessed differences between participants.

### 2.4. Ethics Approval

This study was reviewed and approved by the ethics committees of Asociación Benéfica PRISMA in Lima, Peru (CE2601.15), and the Johns Hopkins Bloomberg School of Public Health in Baltimore, USA (IRB00073874).

## 3. Results

### 3.1. Participants

We conducted a total of 31 in-depth interviews with participants from 11 different communities. In Table 1, we describe the types of informants and number of interviews conducted with each informant type. Of the primary cooks and family members interviewed, three used only a traditional stove, one used both a traditional and locally improved stove, four used both a traditional and LPG stove, one used both a locally improved and LPG stove, and two used a traditional, locally improved, and LPG stove.

Of the interview participants, 9 of the 31 were also observed preparing different meals: four breakfasts, three lunches, and two dinners. Observations were conducted with one household using only a traditional stove, two using only a locally improved stove, three using an LPG and a locally improved stove, two using a traditional and an LPG stove, and one using a locally improved, traditional, and LPG stove.

### 3.2. Key Themes

Six key themes emerged from the data as important factors influencing the adoption and exclusive use of clean cooking:

1. Perceived differences in food taste and nutrition by stove type
2. Cooking niches filled by different stoves
3. Social norms related to cooking practices
4. Safety concerns
5. Comparative costs of buying and using different stoves
6. Limited perceived connection between stove use and health

#### 3.2.1. Taste and Nutrition

Nearly all participants noted that the taste of food is different depending on the type of stove used to cook. All participants expressed a preference for food cooked on biomass-burning stoves (locally improved or traditional), stating that the flavor of the food cooked on these stoves is much better or “tastier” than food cooked on LPG stoves.

“In the traditional stove the food has more flavor, in gas stoves the food doesn’t have flavor … like the food is sour in gas stoves.”

—Family member, male, traditional and LPG stoves

Some individuals described the taste of food from an LPG stove as “different”, while others described the taste as “unnatural”. However, when speaking about the taste of food from biomass-burning stoves, participants frequently used the word “delicious”.

“My brothers cook with gas and it doesn’t have flavor, the *caldo de patasca* (corn and tripe soup) has another kind of flavor, it isn’t very delicious.”

—Family member, male, traditional stove

Some individuals attributed the taste difference between LPG and biomass-burning stoves to the different pots used for cooking on each. When cooking on biomass-burning stoves, primary cooks use customary, traditional clay pots; however, while cooking on LPG stoves, primary cooks believe they need to use aluminum or metal pots that can be placed on a metal surface without splitting.

“Some people say that gas cooked food doesn’t have any taste … We are using pots made of clay and you can’t use it to cook with gas, it has to be metal or aluminum. We cook in clay pots and the taste is just delicious, in (locally) improved stoves we can use those pots.”

—Health worker, female

Others believed food tasted different based on the fuel source, stating food cooked with cow dung or wood tastes better than food cooked with LPG.

“Cooking with cow dung is more delicious, with gas it is easier to cook but it is not delicious—it doesn’t have a good flavor.”

—Primary cook, female, traditional and LPG stoves

Many people also mentioned that the fuel and pot used influence the nutritional content of the food, including one doctor who explained that clay pots and smoke from biomass-burning stoves give food more nutrients than LPG or aluminum pots.

“(LPG) makes food taste unnatural, but wood doesn’t. Wood makes food have all its properties, proteins, minerals, and such.”

—Doctor, male

#### 3.2.2. Cooking Niches

Participants noted that LPG stoves, locally improved stoves, and traditional stoves are useful in different situations. LPG stoves are often used when an individual or family is in a rush or wants to warm food quickly. This can occur when people are getting ready for school or work or return home late in the evening. People also tend to prefer to use LPG in bad weather, such as rain or wind, when wet biomass fuels are hard to light and gusts extinguish the flames.

“We use the (LPG) stove when we are in a hurry. The wood stove won’t start that quickly, and it takes time to get hot enough…wood takes time to catch fire.”

—Family member, female, traditional and LPG stoves

In addition, it is common for individuals to see LPG stoves as a convenience tool, for instance at night when they are feeling “tired” or “lazy”. LPG stoves are also often used when food preparation is viewed as an “emergency”, such as when a family receives unexpected guests or a family member is ill. Participants generally reported using LPG when they want to save time.

“In the villages we use gas stoves in emergencies, that’s why we bought it. Now we cook faster. I have to go to school, that’s why I have to cook faster so I have the gas stove.”

—Family member, male, traditional and LPG stoves

A stove’s ability to generate and sustain heat was another major factor influencing stove choice. Participants noted that they appreciated the warmth from biomass-burning stoves, especially in the winter and early morning when temperatures can be very cold in Puno.

“When we talk about this (warm) season it’s okay to cook with gas. But when it comes to the frost season you cook with wood (because) it also works as heating, so people would rather use wood as the temperature gets really low and it’s terrible. When you use gas to cook, your kitchen doesn’t get warm, but when you use wood, your kitchen turns very hot.”

—Community leader, male

Other participants liked that the embers in biomass-burning stoves keep could maintain the heat of food after cooking.

“The gas stove lets food get cold faster, but in the traditional stove, (the food) keeps warm longer than in the gas stove, that’s why I cook (on the traditional stove).”

—Primary cook, female, traditional and LPG stoves

Many people perceived the heat from their locally improved stove to be stronger and hotter than the heat from their traditional stove.

“I prefer (the locally improved stove) in the afternoons because it burns hotter and the fire doesn’t turn off fast and it makes the food faster. Also, when I boil the water it is hot until the morning and doesn’t get cold fast.”

—Primary cook, female, traditional, locally improved, and LPG stoves

#### 3.2.3. Social Norms

Traditional cooking practices are highly ingrained and embraced in this setting, where living traditionally is valued. When time is available, many of the primary cooks stated that they were willing and even enjoyed spending their available time collecting fuel and cooking. These tasks were generally viewed as normal chores, and were often described as valued social opportunities.

A few individuals expressed frustration with how quickly the LPG stove heats food because they are not comfortable cooking at that pace. Some also reported that it is not possible to prepare certain traditional dishes on LPG stoves.

“*Thimpo de trucha* (fish soup), we have to cook it slow (with the *fogón*) so it gets a better flavor… (Also), we can’t toast the *tostado* (corn nuts) in a gas stove, we have to toast it in a *fogón*.… On the traditional stove we can make *mazamorra de quinua* (quinoa porridge), which can’t be made on a gas stove either.”

—Community leader, male

Participants also mentioned that the three-burner traditional stove is more suited for cooking large family meals. Given that many locally improved stoves are also made with three burners, participants have minimal trouble adapting. However, participants noted that cooking large quantities on a two-burner LPG stove is challenging and requires changing their cooking routines.

“This (locally improved) stove is to cook for a lot of people. My nephews sometimes come from Puno (the city)… I have to cook in big pots because they come from Puno. On the gas stove it takes more time to cook.”

—Primary cook, female, locally improved and LPG stoves

Though biomass-burning stoves were popular in this population, both LPG users and non-users also described some benefits of LPG stoves, indicating a potential willingness to adopt or more consistently use LPG stoves despite prevailing norms. For instance, all participants acknowledged that LPG stoves are cleaner than both traditional and locally improved stoves. Some focused on how their pots or kitchens stayed much cleaner when using LPG stoves. Others made reference to their hands being cleaner when using a LPG stove because there is no need to touch wood or animal dung.

“When using gas it is cleaner. It’s easier to clean than the *fogón* which gets your hands dirty.”

—Community leader, female

#### 3.2.4. Safety Concerns

Many participants noted their own or their community’s fear of LPG stoves. Overall many view LPG stoves as dangerous, and fear that they will explode or cause fires.

“This gas-based stove is dangerous. …I don’t want to use the gas always.”

—Primary cook, female, traditional, locally improved, and LPG stoves

Some participants perceived that their children and younger people in general do not have the same fears and actually prefer to use LPG.

“…this (LPG) stove…scares us since it is dangerous. We don’t want to use it. The kids like to use it; they are currently the ones that use gas stoves.”

—Family member, male, traditional and LPG stoves

Although none of the participants had experienced an explosion or incident with an LPG stove and had not heard of an incident happening in their community, they feared the possibility.

“They think it could start a fire, so they are afraid of it...nothing like that has happened [here]…, but I’ve heard that in other places there have been fires because of the gas, so the elderly are scared…there could be a gas leak that starts a fire.”

—Community leader, male

#### 3.2.5. Cost Perceptions

All participants noted that the high cost of locally improved and LPG stoves is a barrier to adoption. According to the local contractors interviewed, LPG stoves cost roughly S/. 70 (approximately 21 USD) and can last for 10 years; however, refilling the LPG tank is seen as a substantial household expense, costing most households roughly S/. 36 (11 USD) every two weeks if the LPG stove is used regularly. Locally improved stoves cost a family between S/. 400–700 (120–210 USD) to build and last five to seven years (though all of the participants in this study who owned an improved stove received it for free through the government programs mentioned above). In comparison, traditional stoves require an investment of approximately S/. 18 (5 USD) every two to four years for stove construction or maintenance.

Most participants preferred to save money by gathering their own fuel for use in the traditional or locally improved stoves, despite the additional time burden. Since all stoves require an upfront payment but only LPG stoves require continuous payment for fuel, initial stove costs were perceived as more acceptable than continuous payments for LPG fuel refills.

“The gas…it is expensive! I heard that there are some people who sell it for 20 soles, but still I do not have (the money) for that.”

—Primary cook, female, traditional, locally improved, and LPG stoves

Though the government provides vouchers that reduce the price of one 20 kg LPG tank containing 10 kg of LPG by S/. 16 (5 USD) every month, many households opt not to redeem the vouchers because they still must contribute approximately S/. 20 (6 USD) to purchase the LPG tank.

“We get the tickets to buy gas but we don’t buy it because we would do it just for the sake of it. We’re benefited every month and we don’t take it.”

—Family member, male, traditional stove

For poor households, simply feeding the family takes priority over paying for LPG or building a locally improved stove. Though community leaders viewed the uptake of new and improved stoves as important, they generally reported that electricity, plumbing, and water were higher priorities. However, when focusing on cooking, most leaders believed that the locally improved stove is the most affordable and sustainable choice for their own home and the community.

#### 3.2.6. Limited Health Awareness

People do not generally see a strong connection between stove use and health. In most interviews, participants did not mention health as either a benefit of locally improved stoves or LPG stoves, or as a drawback of traditional stoves, until the interviewer specifically asked about it.

Interviewer: “Does the stove affect your health?”

Interviewee: “Well, yes, earlier (the traditional stove) affected me, but now I don’t think it affects me because we are cooking on the (locally) improved stove.”—Primary cook, female, traditional and locally improved stoves

Of all study participants, the contractors who build locally improved stoves put the most emphasis on the negative health effects of traditional stoves:

“I think that the stoves we make are a way to improve families’ lifestyles so they won’t have to live with the smoke they get now; it causes illnesses. Families will live better, free from smoke, they won’t cry because of it.”

—Contractor, male

Many of the health care workers interviewed said that most people in the community do not value prevention, but instead focus on treatment after a health problem occurs. This could contribute to the fact that people generally did not view the longer-term health impacts of household air pollution exposure as a problem. Instead, people valued the short-term effects of smoke reduction (i.e., reduction in coughing or elimination of teary eyes).

“Promoting health prevention is a little abandoned. We don’t take prevention seriously, and patients don’t…have an optimal idea of what prevention is. If you tell a patient to drink water to improve their health, you are a bad doctor. If you give them lots of pills you are a good doctor.”

—Doctor, male

A few individuals reported that they were not worried about smoke at all in relation to their health, while others stated that smoke only affects women and children who are perceived to be more vulnerable. A few stated that LPG stoves are worse for health than traditional stoves due to the smell of the LPG or the “fumes” it produces. Although reducing exposure to smoke was not a top priority for most participants and not a priority at all for some, people did recognize that the locally improved stoves produce less smoke than traditional stoves. Although LPG stoves also eliminate smoke in the home, locally improved stoves were more frequently praised for this improvement. Generally, people appreciated that locally improved stoves reduced smoke in their home while allowing them to continue cooking in the same way they did with their traditional stove. Even people who did not own a locally improved stove at the time of the interview referenced smoke reduction or elimination as a top reason for wanting one.

## 4. Discussion

The results of our study suggest that adoption of clean cooking is influenced by many factors unrelated to health, which must be considered and addressed by programs aiming to increase use of clean cooking technologies. While technological advancements in stove and fuel performance are necessary to ensure that technologies are clean, efficient, and meet user needs, promoting behavior change among users to adopt such clean technologies is also important for achieving the WHO IAQ targets. This study has demonstrated that efforts to promote sustained and exclusive adoption of clean cooking technologies must address user preferences about when and for what cooking tasks different stoves should be used, the social and cultural norms related to cooking practices, the benefits of clean cooking that are most highly valued in the community, beliefs about the taste of food cooked over different fuel sources, as well as the affordability of clean cooking technologies and fuels.

As many studies have found [12], participants in this study commonly reported stove stacking, choosing different stoves for different cooking tasks. Given that just one hour of traditional stove use per week can increase indoor air pollution levels above the interim WHO IAQ target [6], the findings from this study can facilitate the design of technologies that better meet user needs and implementation of behavior change strategies that will best encourage exclusive use.

One potential behavior change strategy for promoting acceptance and use of clean cooking is to encourage trusted community leaders and elders to become “early adopters” of clean cooking. The leaders interviewed in our study shared many of the same opinions as the other community members interviewed, but because of their respect and influence it is likely that convincing them to adopt LPG could convince other community members to do the same [22]. To overcome the prevailing gender norm noted in this study that it is part of a woman’s role to spend long periods of time cooking, it may be necessary to emphasize clean cooking attributes that appeal to both men and women [11].

Some projects have attempted to address negative perceptions of improved stoves by using cooking demonstrations, which aim to encourage stove uptake by allowing people to observe firsthand the process and benefits of cooking with an improved stove [11]. A study in Uganda found that cooking demonstrations were the most effective strategy for promoting purchase and correct use of an improved stove [23]. However, there is a lack of information on how cooking demonstrations affect adoption beyond the initial purchase phase. Our study suggests that it could be useful to include targeted behavior change messaging in cooking demonstrations, addressing key barriers and motivators influencing household adoption of LPG stoves. These messages could highlight that (1) nutrients come from the food ingredients not the pots or the smoke, (2) smoke is far more dangerous than LPG fumes, (3) LPG will keep your home, pots, hands, and clothes cleaner, (4) cooking with LPG is fast and gives you more time for other activities, (5) LPG stoves can be used under all weather conditions, (6) LPG stoves are safe when used correctly, (7) all tasks done with a traditional stove can be done with LPG, and (8) LPG will eliminate smoke in your home. Demonstrations can also be an opportunity to educate users not only on how to operate the stove safely, but also on how to prepare food at the fast pace allowed by LPG, how to cook large meals with only two burners, and how to spend the time saved from faster cooking (i.e., spending more time with family or friends, or doing other income-generating work). Additionally, given the many fears about LPG raised in our study, ensuring that safety regulations for the LPG industry are sufficient and transparent will be essential for alleviating consumer concerns.

There is a growing body of literature suggesting that long-term health benefits may not be as valued by potential improved stove users as other stove attributes [11]. In our study, people reported valuing the smoke reduction provided by locally improved and LPG stoves, but the longer-term health benefits were not major motivators. Participants said they appreciated smoke reductions because they experienced less coughing and watering eyes, and their homes stayed cleaner. This suggests that emphasizing the cleanliness and short-term health benefits of smoke reduction in stove promotion efforts may be more effective for motivating adoption than focusing on longer-term health impacts. Because many people also reported that only vulnerable people such as women and children are susceptible to the ill effects of smoke, it may also be necessary to raise awareness that smoke affects everyone in the household, including men. However, emphasizing smoke reduction and health benefits alone is unlikely to motivate adoption unless paired with other valued benefits.

Preference for the taste of food cooked over biomass-burning fires is frequently cited as a reason that people continue using their traditional stove even after purchasing a cleaner stove [5,24]. Similarly in this study, people reported that food tastes better when it is cooked over a biomass-burning fire. Whether due to the difference in type of pot (clay vs. aluminum), or the belief that smoke adds nutrients, there is a prevailing perception that food cooked with LPG lacks flavor. However, Bhojvaid et al. [24] found that taste was mainly an issue for older people who had rarely left the village, with more worldly and well-travelled people being more accepting of the taste of food cooked with LPG. This suggests that targeting younger people in communities and engaging them to promote clean cooking as a new and modern norm could increase acceptance of food cooked with clean technologies.

Because cooking is a habitual behavior that is performed multiple times every day, new habit formation is an essential step for fostering adoption of clean cooking behaviors and overcoming preferences for traditional stoves. Habit formation involves performing behaviors repeatedly over time, with a satisfactory result in a stable context [25]. This implies that for adoption of clean cooking behaviors to occur, people must repeatedly use the stove, be satisfied with its performance, and have stable access to stoves and fuel. This habit formation could be promoted through visual cues to action, such as positioning the improved stove strategically in the cooking area, hanging reminder signs, and posting pictures detailing how to use the stove. Such pictures would also improve self-efficacy for performing the behaviors and increase the ease of use, which have also been shown to promote habit formation [26]. Also, since forming new habits may be most feasible at times of major life changes [27], framing LPG stoves as ideal wedding gifts or gifts for the birth of a first child might be an effective strategy for encouraging adoption among young couples. However, the most appropriate occasions for such gifts and whether a LPG stove would align with gift-giving norms would need to be investigated.

Our study also suggests that although financing is important, addressing cost alone is not enough to motivate sustained adoption. Specifically, even though LPG is highly subsidized by the Peruvian government, participants were still reluctant to make use of the vouchers to obtain and use LPG fuel given other non-cost related cooking perceptions and preferences. Still, comparing costs associated with different cooking methods can be an important component influencing LPG stove uptake. For example, highlighting that LPG stoves cook quickly and reduce the need for fuel thus providing people with more time for other income-generating tasks, and that LPG stoves can improve health thus reducing health care costs, might help motivate adoption when combined with other behavior change strategies.

In cold climates such as Puno where people appreciate the heat produced from biomass-burning fires, it may be necessary to offer alternative methods of heating to fully displace the use of fires. Though this was not a primary reason for using the *fogón* in Puno, more work should be done to investigate the feasibility and effectiveness of heating strategies such as area heaters powered by LPG or electricity [7], solar heaters [28], or promoting the use of clothes and hot drinks to stay warm. Given both the heating capacities of biomass-burning stoves as well as their closer compatibility with current cooking practices, manufacturing and promoting improved biomass stoves may be a more feasible approach in cold communities where biomass is available free of charge.

### Strengths and Limitations

This study was limited by the fact that data were collected primarily during the winter season. Due to the colder temperatures during this season, people may have focused more on issues such as heating and poor weather than if interviews had been conducted in the warmer summer season. However, given that temperatures are still cool even during the summer season, and rain and wind remain common throughout the year, similar concerns would likely exist year-round. Additionally, the data collectors were employed by a local organization that provided locally improved stoves to many people in the community where interviews were conducted. People may have been reluctant to express negative views about the locally improved stoves, or may have over-stated their dislike of alternative stove models, in an attempt to please the data collectors. In an attempt to mitigate this concern, data collectors were instructed not to emphasize their association with the organization and to inform participants during the consent process that the study would maintain strict anonymity and confidentiality. Additionally, our study did not explore the process of decision-making for LPG stove purchase and use, including the potential impact of gender dynamics and how these decisions may change over time. We also did not interview LPG stove manufacturers and distributors. However, by triangulating findings across different types of participants and data collection methods, contextualizing reported information with observational data, and collecting rich descriptions of factors influencing stove preferences, this study provides credible evidence that corroborates and expands upon existing knowledge related to clean cooking adoption.

## 5. Conclusions

Although we have proposed many potential behavior change strategies for increasing adoption of clean cooking technologies, more research is needed to determine the relative impact of the different strategies. There is a need for more behavioral-focused randomized trials to test the impact of behavior change strategies on adoption of clean cooking. For example, determining if the following interventions would encourage more widespread adoption are areas for further inquiry: (1) using early adopters to promote clean cooking, (2) conducting cooking demonstrations, (3) targeting younger generations, (4) holding blind taste tests, (5) providing cues to action, and (6) marketing LPG stoves as gifts for major life events. Additionally, given our findings that financing schemes alone are not sufficient for encouraging clean cooking adoption (as evidenced by the fact that some people did not exchange their LPG vouchers), further investigation into how financing schemes can best be paired with behavior change strategies to achieve exclusive and sustained adoption are necessary. More research is needed to understand the factors beyond cost that influence use of clean cooking technologies. Despite increasing awareness of the importance of uptake and sustained adoption of clean cooking for reducing household air pollution, more work is needed to test the impact of different behavior change strategies on longer-term adoption. While factors influencing adoption may vary slightly across countries and regions, the barriers and motivators identified in this study and others can inform the development of successful behavior change approaches to advance clean cooking adoption globally.

## Figures and Tables

**Table 1:** Number and type of participants included in the in-depth interviews.

Participant Type	Total
Primary Cooks	
Female	6
Family Members of Primary Cooks	
Male	4
Female	1
Health Workers	
Doctor	1
Nurse	1
Nurse Tech	2
Promoter	2
Community Leaders	
Lieutenant	3
President	4
Mayor	1
Secretary	1
Improved Stove Contractors	
Boss	2
Employee	3
Total	31
